# Notes and Comments

**Published:** 1924-02

**Authors:** 


### NOTES AND COMMENTS.

### THE NEW MINISTER OF HEALTH.

THE hazards of politics have now added yet another to the already long list of Ministers of Health. Mr. John Wheatley, the member for the Shettleston Division of Glasgow, is the least experienced and the most untried of the series, and he goes to Whitehall with the reputation of being one of the most extreme representatives of Socialism. So long, however, as he makes a good Minister of Health few people will trouble about his politics. It will be surprising if Mr. Wheatley does not, in great measure, concentrate upon housing, which is a peculiarly sore subject on the Clyde. Yet, in view of the remarkable statement made by his predecessor in the debate on the Address, it is hard to see that much more can be done than is being done. The new Housing Act has been in operation less than six months, yet its success is so considerable and, it may be added, so unexpected, that 100,000 houses will be built under it this year. That is above the annual pre-war average. What is now needed is not fresh legislation, but a larger number of skilled building operatives, and Sir W. Joynson-Hicks declared that still more houses could be built if more skilled labour were available. And it is important to observe that the great majority of these dwellings are being built by private enterprise.

### HOSPITALS AND POOR LAW INFIRMARIES.

FOR some time past our columns have been bearing witness to the growing feeling that the time has come when arrangements ought to be made whereby the half-empty Poor Law Infirmaries may come to the rescue of the crowded hospitals. Mr. E. W. Morris, the House Governor of the London Hospital, has been addressing the Guild of Pharmacists on the subject, and an extensive summary of his stimulating speech will be found elsewhere in our present issue. On the face of it, it is grotesque that the London should be more than full while the Whitechapel Infirmary has many empty beds. Such glaring lack of co-ordination and of failure to make the best of what we already have justifies the suspicion that we are not always quite such a practical, commonsense people as it pleases us to suppose. The Boards of Guardians have 92,000 beds at their disposal, yet some two-thirds of them are usually empty. The principal reason for this heavy percentage of disuse is that those who enter the Poor Law Infirmaries have hitherto been classed as paupers. The stigma is archaic and indefensible, but its existence sends to the hospitals many patients who would be well cared for in the Infirmaries, which have reached a high standard of excellence in equipment and nursing. Sick persons not belonging to the class for which the Infirmaries were primarily intended may now resort to them upon payment of the cost of their treatment, and Mr. Morris suggests that patients should be sent to them from the hospitals, whose consultants would visit them, while their qualified students should act as house surgeons and physicians under the Infirmary medical officers. These patients would be segregated and wear their own clothes. Nobody would think of them as paupers, while the hospitals would be relieved of cases that are not urgent or especially difficult in favour of those that are. The voluntary hospitals contain only one third of the beds they need, or very soon will need, while the Infirmaries contain two-thirds more than they need. The conclusion is irresistible, and nothing that the new Minister of Health could do would be more practically valuable or more welcome to the patiently waiting sick than the removal of an anomaly which is as cruel as it is absurd.

### THE PUBLICITY OF INQUESTS.

DR. WALDO, the Coroner for the City of London, has done good service by calling attention, in his Court, to the importance of giving publicity to inquests. The " Crowner's Quest " is one of the most ancient of our institutions, and in the City its functions are not confined to inquiries into the cause of uncertificated deaths, but extend to investigations into the origin of fires. Dr. Waldo is properly jealous for the importance of these Courts, and anxious that they should be conducted with the fullest regard to publicity. He gives notice to the Press and the public of every inquest held by him, and he has expressed the opinion that this course should be made compulsory upon all coroners. He is a stickler for juries—now so often omitted at inquests—especially in cases of suicide. Juries are direct representatives of the public and are an obvious safeguard. In inquests into suicides, letters left by the unhappy victims are sometimes read in court and published in the newspapers, where they may easily do harm to persons upon whose ill-balanced minds they may act with all the force of suggestion. If they are seen by the jury and the relations all the purposes of publicity are served. The plea that it is absurd to take busy men away from their work to attend a " trumpery inquest " is not very impressive; a coroner's jury is usually detained for a very short time, and the number of people with no pressing business to attend to is considerable.

### THE WEATHER AND SICKNESS.

PROBABLY, at one time or another, every sort of weather has been blamed for every sort of sickness. The connection is occasionally so obvious that popular generalisations and exaggerations are bound to follow. Nevertheless there are such things as indirect weather effects which, though we may smile at them, have a very real meaning in everyday life. The Editor of the *Long Island Medical Journal* has been collecting examples, and some of these are most interesting. He quotes, for example, the tendency of pneumonia patients to do badly if their crisis occurs during a storm and the fact that the temperature will often rise in neurotic people at the advent of a thunder shower. The story is told of an old sea captain in Charlestown who had a great reputation as a weather-prophet, but would never give away his secret until, late in life, he admitted that he could foretell a storm by the onset of severe aching in a corn. We all realise, of course, that deaths are more frequent on very hot or very cold days; the general strain on the system is increased. There is no doubt, too, that a coming storm has a definite psychological effect on both men and animals. The old belief that rains caused malaria is easily explained when we remember the life-history of the parasite concerned. Therefore, we must hesitate before we laugh to scorn some of the sayings of old. The influence of weather on sickness is very real, though it may not really act quite in the ways that our forefathers believed.

### THE "LONDON'S" LATEST ROMANCE.

THE London Hospital is a very romantic institution, and from the Whitechapel Road, as from Africa, there is always something new. The newest thing that has happened to the "London," and perhaps the most romantic thing that has ever happened to it, is the sudden acquisition of an endowment fund of £180,000, producing an annual income of £8,500 a year. An anonymous benefactor offered to give any sum up to £80,000, provided the public would give pound for pound by a certain date. The public gave over £90,000, and when the prescribed limit was reached Sir H. Mallaby Deeley promised to double the surplus up to £10,000. This is a magnificent and, withal, exceedingly touching, since no inconsiderable sum came from the manual workers of the East End and other givers of very small sums. Thus the abounding sympathy and unstinted help which the London Hospital gives to a wide district has been heartily repaid. It will still, of course, need money, but it will be greatly helped in meeting its ever-increasing liabilities.

### HUMANE SLAUGHTER.

WERE it not that it is eternally true that "errors, like geese beheaded, run about," we might reasonably hope, in view of the experiments recently made at Birmingham, to hear little more of the brutal old method of slaughtering cattle with the pole-axe. A public demonstration, designed to show the superiority of that weapon over the various humane killers, was organised at the Birmingham abattoir by the National Federation of Meat Traders' Associations, but of the two bullocks slaughtered in this way one remained standing after the first blow. On the other hand, the five who were slaughtered by mechanically-operated humane killers were rendered insensible by the first shot; and while nine pigs were successfully shot by various types of humane killers, four blows with the hammer were needed to stun the first pig. The joint report, which has been issued by the Federation Defence Society, condemns the Jewish method as "primitively barbaric" in that it is "essentially opposed to depriving the animals of conscious feeling before the knife is applied." It appears that in London a thousand bullocks are slaughtered every week by this dreadful method, but since religious tenets enter the matter it is probably hopeless to expect reform, save in cases where the meat is not actually intended for Jewish consumption. Speaking generally, however, now that the superiority of humane killers has been conclusively proved, there ought to be no serious delay in abandoning the cruel methods we have inherited.

### LEGACIES TO HOSPITALS.

TWO years and a-half ago Lord Cave's Committee recommended that bequests to hospitals should be exempt from the 10 per cent. legacy duty on the ground that "there appears to be no good reason why the State should intercept at the source one-tenth of all sums bequeathed for the benefit of the sick." Nothing, however, has been done towards honouring this recommendation. When an attempt was made in Parliament to give effect to it, it was objected that, since bequests of this kind are usually given free of duty, it would be the residuary legatee, and not the hospitals, who would benefit, and that it would be difficult to resist similar claims from other voluntary philanthropic organisations. This latter objection may be dismissed as destitute of substance, since hospitals are in an entirely different position from all other forms of charitable effort. Out of the other difficulty the Lincolnshire Voluntary Hospitals Committee suggest a simple and feasible way of escape. They propose that the duty should still be collected by the Treasury and then transferred to the Voluntary Hospitals Commission for distribution among necessitous hospitals according to regulations officially approved. If this solution were pressed upon any Government we should not be surprised if it were objected that the Treasury could not afford to lose the money, which the Lancashire Committee estimates at about £100,000 a year. Such an arrangement, however, while merely taking from the Exchequer money to which it has little moral right, would afford exceedingly valuable help to the hospitals at a time when they are sorely in need of help. We must not forget that formidable arrears of building and equipment have accumulated, and that, with the means at present available, there is little opportunity of doing work which becomes more urgently necessary every month.

### FIGHTING TUBERCULOSIS OR THE TUBERCULOUS?

AN excellent example of how to make tuberculosis a subterranean disease to which no one will confess till he is dying, is to be found in the "Rules and Regulations for the Control of Tuberculosis," put into force since January 1, 1923, by the Department of Public Health in Illinois. It appears from these Rules that any person having knowledge of a suspected case of tuberculosis must notify it to the Local Health Authority. In England this duty is incumbent only on the medical practitioner. But in Illinois it is incumbent on any and everyone to smell out cases of tuberculosis and report them. Again, a husband may not sleep in the same room as his wife in Illinois if he or she is suffering from "open" tuberculosis. The patient who does not comply with these and other regulations is liable to have his house conspicuously placarded so that public ostracism may drill him into the desired state of docility. If all these measures could be so administered as to hit the tubercle bacillus and not its host, no one would complain. But so long as legislators think only in terms of bacilli and ignore the human element they are likely to be the best friends of the tubercle bacillus. The secret of success in the campaign against tuberculosis is early diagnosis and early treatment. So long as the tuberculous are hounded out of the community like a pack of lepers, the person developing early tuberculosis is not likely to take the steps necessary to bring about the early recognition of his complaint. In helping to achieve an early diagnosis the prospective consumptive will show about as much vivacity and alacrity as a moulting hen, and who shall blame him so long as the health authorities are blind to the elementary fact that stamping on the patient is not the best means of stamping out the disease ?

### WHITE LEAD.

ON the first day of the present year the White Lead Convention prepared by the International Labour Conference at Geneva came into force in the countries which have adhered to it. England is not one of those countries, Parliament having refused to ratify the Convention. In addition to laying down certain regulations for the use of lead paints, the Convention prohibits, with specified exceptions, the use of more than 2 per cent. of lead for interior painting after November 19, 1927. One reason which influenced the late Government in their opposition to ratification was probably the fear of adding to unemployment by reducing the amount of work available in the British lead industry. Of late, however, there has been a large increase in the production of zinc oxide, which is free from the perils of white lead, and it would not be unreasonable to expect that when the manufacture of zinc oxide is sufficient to make good the decreased production of lead, the Convention should at last receive ratification. That the dangers of white lead are serious is notorious, and some statistics recently issued by the Home Office show that in five years more than a thousand cases of lead poisoning, with 169 deaths, came to the knowledge of the Factory Department. Yet, since house-painters are outside the Factory Acts, there must have been a much larger number of cases of which there is no record. The importance of seriously reducing industrial diseases is so great that we trust that the subject will not be allowed to slumber for long.

### IS APPENDICITIS CATCHING?

DR. A. FONIO, of Switzerland, has added a new terror to appendicitis by suggesting that it is an infectious disease. Were his suggestion merely an arm-chair after-dinner speculation, unsupported by tangible evidence, he could be dismissed forthwith as a scaremonger. But he has marshalled an impressive array of facts in support of his theory. He has conducted inquiries among the 275 patients on whom he has operated for appendicitis, and has found that in the immediate neighbourhood of 151 of these patients there had been one or more other cases of appendicitis, the disease being diagnosed by some competent physician or surgeon. There were altogether 245 such " contact cases " of appendicitis. Now, had Dr. Fonio checked these investigations by selecting at random 275 healthy persons and found out how many cases of appendicitis had occurred in their immediate neighbourhood, he would have strengthened his case greatly if he could have shown that there were hardly any cases of appendicitis in contact with the healthy controls. But he has not taken this precaution. Therefore those who cling to the view that appendicitis is not infectious may point out that, this disease being very common, all Dr. Fonio's " contact cases " were nothing more than coincidences. We do not, for example, argue that fractures are infectious because two or three members of the same family break a bone. Dr. Fonio has, however, followed another line of research, and his examinations of appendices removed in the early stage of appendicitis have brought him the conviction, or at least the strong suspicion, that there are one or two special germs which have a peculiar and sinister affinity for the appendix. If bacteriologists confirm his findings, we may look forward to a time when prophylactic vaccination against appendicitis will be as fashionable as vaccination against smallpox and typhoid fever. If this prophecy comes true, we may live to see a slump in abdominal surgery.

### HOSPITAL BALLOTS OLD AND NEW.

IN these days of revived interest in raffles and ballots on behalf of hospitals, it is interesting to recall the strange adventures that befell the founder of the Rotunda Hospital, Dublin, when resorting to them. Sir William Smyly lately retold the story, which is one of the most curious in hospital history. In 1745 Dr. Bartholomew Moss determined to build the Rotunda Hospital, but he had no money and the estimate was £20,000. He started by giving entertainments in the gardens at the back and then organised a big sweepstake for which thousands of tickets were sold in England. Dr. Moss visited London to attend the drawing, only to find that the agents for the sale of the tickets had bolted with the money, leaving him without funds for his hospital and responsible for the prizes. Since he could not meet these claims, he also bolted, was arrested at Holyhead and imprisoned at Beaumaris. He escaped and remained in hiding in Anglesey for a time. At last he returned to Dublin, when the Irish Government of the day paid his debts. This was an unexpectedly happy ending, but the lesson is worth remembering, and that is why it is so necessary to have an unimpeachable authority responsible for any ballots that may be held for charity. When held at all, they are held in numbers, but, like roller-skating, they seem to have a periodic, but intermittent, popularity.

### BLOOD TRANSFUSION.

THE " giving of blood " is gradually becoming something more than the mere resuscitation of a failing constitution by the introduction of fresh healthy blood. In recent numbers of the *Lancet* there appear records of further successes at St. Mary's Hospital with the transfusion of human blood elements, the resistance of which has been artificially raised against the bacterial infection which is attacking, possibly killing, the patient. In this way the patient's circulation is directly supplied with artificial powers to overcome the infection and later to help itself back to health. The healthy donor is first inoculated with the germ or " vaccine "

of the patient's infection, and when the former's resistance is raised and his blood has acquired sufficient artificial immunity, some of it is withdrawn and slowly injected into the patient's veins. Some remarkable recoveries of previously desperate cases are recorded.

### THE OPEN-AIR NURSERY SCHOOL.

SOME valuable pioneer work is being done by the Nursery School Association which was formed recently to ensure that the clause in the 1918 Education Act providing for schools of this kind should be effectively carried out. At present these schools are few and, says Miss Margaret McMillan, the President of the Association, from 200 to 300 are required in the next few years, and required not only in London but in Birmingham, Manchester, Leeds and Bradford. As to the question of sites, Miss McMillan states that they are available in the East End. A small body from each local authority is needed to make a survey of the worst and most crowded districts. The school buildings are, of course, not elaborate. A number of open-air shelters constitute a series of little schools under one management, and the school should serve a two-fold purpose—a place of training for young children and a place for recreation for young mothers. Educated women are needed as workers in the schools where children are received at the age of two years and where in addition to learning how to live healthily they are to learn to speak good English. The Association's chief aim, and it is one which deserves support, is to impress on public authorities the necessity for nursery schools being incorporated in our educational syst m. If this were done the elementary schools would eventually be the gainers, since they would receive pupils in an infinitely higher state of physical and mental development than is the case at present.

### OVERHEATED ROOMS.

THE editorial columns of the *Journal of the American Medical Association* recently reviewed the possible ill-effects of overheated rooms. In view of the widespread adoption of central heating in America, and its increasing use in this country, the American findings are of particular interest. It is often held that, provided the air is kept moving and ventilation is good, rooms may be heated to almost any extent without doing harm. In fact, popular praise of central heating seems to assume this point at the outset. If the rooms are not made too hot, it may perhaps be correct, but American experience sounds a definite warning against overheating, the ill-effect of which cannot be balanced by any degree of simple ventilation. They suggest that the evil effects of " bad air " are very largely due to its high temperature, and that 75° F. will produce lowering of natural bodily functions. Many observers have found, however, that if the temperature be inevitably high, their capacity for mental and physical work will be much less impaired if the relative humidity is not high as well. The New York Commission find even 86° F. to be compatible with good work if the humidity is about 80 per cent. In short, if the amount of moisture in the air is kept at the correct level the effect of overheating is less exacting. In too dry an atmosphere, too much water is absorbed from the body and the mucous membranes become dry. In too humid an air, evaporation of perspiration is prevented and bodily depression is inevitable. In the former, active movement of the air would make matters worse; in the latter, it would aid evaporation and improve the conditions. To obtain ideal conditions we must obtain a balance of the various factors, temperature, humidity and air movement. Because windows are open we cannot justifiably make rooms as hot as we like. The question of humidity, moisture and the correct balance of all the factors must be considered.

### INSULIN AND EXPERIMENTS ON ANIMALS.

NOT the least significant passages in the report of the Medical Research Council for 1922–23, which was issued early in January, are those which impress upon the country the means by which insulin was arrived at. That potent agent for the diminution of suffering is the outcome of a long series of experiments on animals, and the service which dogs and other smaller animals are rendering in this way is declared to be much greater than the production of insulin itself can measure. The alleviation of pain and the saving of life are, naturally, the aspects of the matter which fill the public mind and arouse its sense of the romantic and almost the miraculous, but the report declares that it is possible and even probable that the production of insulin " will be found hereafter to have had greater value for medicine as an instrument of research work in physiology and bio-chemistry than in its immediate application to the relief of diabetes." Meanwhile we are now producing insulin in such quantities that we can not only supply the whole of the home demand, but provide a considerable surplus for exportation to the Dominions and to foreign countries which are not yet able to supply themselves. When we reflect that, only a few months ago, this wonderful life-saving agent was scarce and exceedingly dear, we are conscious that we have been witnessing marvels.

### PAPER CONTAINERS AND HEALTH.

AN invaluable aid to public health has been, within the last few years, the adaptation of paraffin wax to paper food containers, and the *Pharmaceutical Journal* has wisely quoted some interesting facts in this connection. Tin may rust and glass may break, but paraffined paper can be, and usually is, absolutely innocuous. If the paper is thoroughly impregnated with the wax so that every pore is filled, it is not only impervious to dirt, insects and all foreign matter, but it is absolutely water-tight. The wax itself is unaffected by salts, acids and alkalis, and its presence actually facilitates the manufacture of some kinds of container. Spraying with molten wax is, of course, a less satisfactory method than complete impregnation of the paper, but in any case the paper container is cheaper than the tin box. For many materials it is hygienically more satisfactory, and one may hope gradually to see its more widespread adoption. One great point in its favour is the ease with which a package can be hermetically sealed; another is the complete absence of taste derived from the container.

